# Supplementary material for: Application of the STRATCANS Criteria to the MUSIC Prostate Cancer Active Surveillance Cohort: A Step Towards Risk-Stratified Active Surveillance
Source: Cancers (Basel). 2025 Sep 17;17(18):3032. doi: 10.3390/cancers17183032 (PMC12468819; doi:10.3390/cancers17183032)
Supplement: Supplementary file 1 [file cancers-17-03032-s001.zip › cancers-3859382-supplementary.pdf]

*Definitions and Classification Criteria*

Post-biopsy covariates were defined within 6 months of the biopsy, with follow-up beginning thereafter in accordance with our verified AS definition. MRI results were either defined as reassuring (PIRADS 1-2), indeterminant (PIRADS 3), or non-reassuring (PIRADS 4-5). For GC, the results were classified as reassuring or non-reassuring based on previously defined MUSIC criteria:

1) Prolaris: > 3% probability of PC mortality
2) OncoType Dx < 80% freedom from primary Gleason 4
3) Decipher Score > 0.45 (indicating higher risk of metastasis and mortality)

*Statistical Analysis*

For our secondary objectives, nine multivariable Cox proportional hazards models were fit, each with cluster-robust standard errors to account for inter-practice correlation. The models adjusted for the following covariates: age, race (White, African American, other, unknown), Charlson Comorbidity Index (0, 1, ≥ 2), family history of PC (yes, no, unknown), and insurance type (private, public, none). Baseline characteristics by STRATCANS tier are shown in Table 1 and 2. Three models were fit for each outcome (upgrading to ≥ GG3, any biopsy upgrading, and time to definitive treatment) with an interaction term between STRATCANS and the prognostic test of interest: (1) pre-biopsy MRI, (2) post-biopsy MRI, and (3) GC testing. Within each outcome, the Benjamini–Yekutieli procedure was used to correct for multiple significance tests of relevant terms. In addition, we plotted hazard ratio (HR) effect sizes from each model, stratified by outcome and test, and normalized to a reference level of no test within each STRATCANS tier. Statistical analysis was performed in R version 4.4.1, with visualization performed using the ggsurvfit package and model fitting conducted with the coxme package [1,2].

*Results*

We assessed whether MRI before or after the diagnostic biopsy, or GC testing, modified the effect of STRATCANS on ≥ GG3 upgrading, any biopsy upgrading, and time to definitive treatment. Pre-biopsy MRI results did not significantly modify the effect of STRATCANS on ≥ GG3 upgrading (interaction term: $p > 0.9$) or any biopsy upgrading (interaction term: $p > 0.9$), but did significantly modify the association of STRATCANS with time to definitive treatment (interaction term: $p < 0.001$). Post-biopsy MRI results did not significantly modify the effect of STRATCANS on ≥ GG3 upgrading (interaction term: $p = 0.3$) or any biopsy upgrading (interaction term: $p > 0.9$), but did significantly modify the association of STRATCANS with time to definitive treatment (interaction term: $p = 0.003$). For GC testing, GC did not significantly modify the effect of STRATCANS on ≥ GG3 upgrading (interaction term: $p = 0.6$), but did significantly modify its effect on any biopsy upgrading (interaction term: $p = 0.026$). GC did not significantly modify STRATCANS' effect on time to definitive treatment (interaction term: $p = 0.096$).

*Interpretation of Forest Plots*

Forest plots displaying the estimated hazard ratios and associated confidence intervals for each combination of STRATCANS tier, confirmatory test of interest (MRI before, MRI after, or GC), and outcome, relative to no test within each STRATCANS tier and outcome, can be seen in Supplemental Figures S1a-c. These forest plots can also examine effect modification in the relationship between STRATCANS and the outcome by comparing effect sizes across different STRATCANS tiers for each combination of prognostic test and outcome. This approach was taken to allow for a clearer visual assessment of effect modification across STRATCANS tiers.

For instance, in Supplemental Figure S1c, for the outcome of time to definitive treatment, large hazard ratios are observed for reassuring and non-reassuring MRIs after diagnosis, with non-proximal point estimates in opposite directions (HR > 1 or HR < 1) and non-overlapping confidence intervals in each STRATCANS tier (relative to no test within each tier). These results align with the significant interaction term for this combination of test and outcome ($p = 0.003$). In contrast, the hazard ratios for reassuring and non-reassuring GC results for the outcome of ≥ GG3 upgrading in Supplemental Figure S1a are all similar, with point estimates close together and highly overlapping confidence intervals, consistent with the non-significant interaction term for this combination of test and outcome ($p = 0.6$).

Supplemental Figure S1.-Forest plots displaying the estimated hazard ratios and associated confidence intervals for each combination of STRATCANS level, prognostic test of interest (MRI before, MRI after, or GC), and outcome, relative to no test within each STRATCANS level and outcome, for (a) upgrading to ≥ GG3, (b) any biopsy upgrading, and (c) time to definitive treatment.

(a)

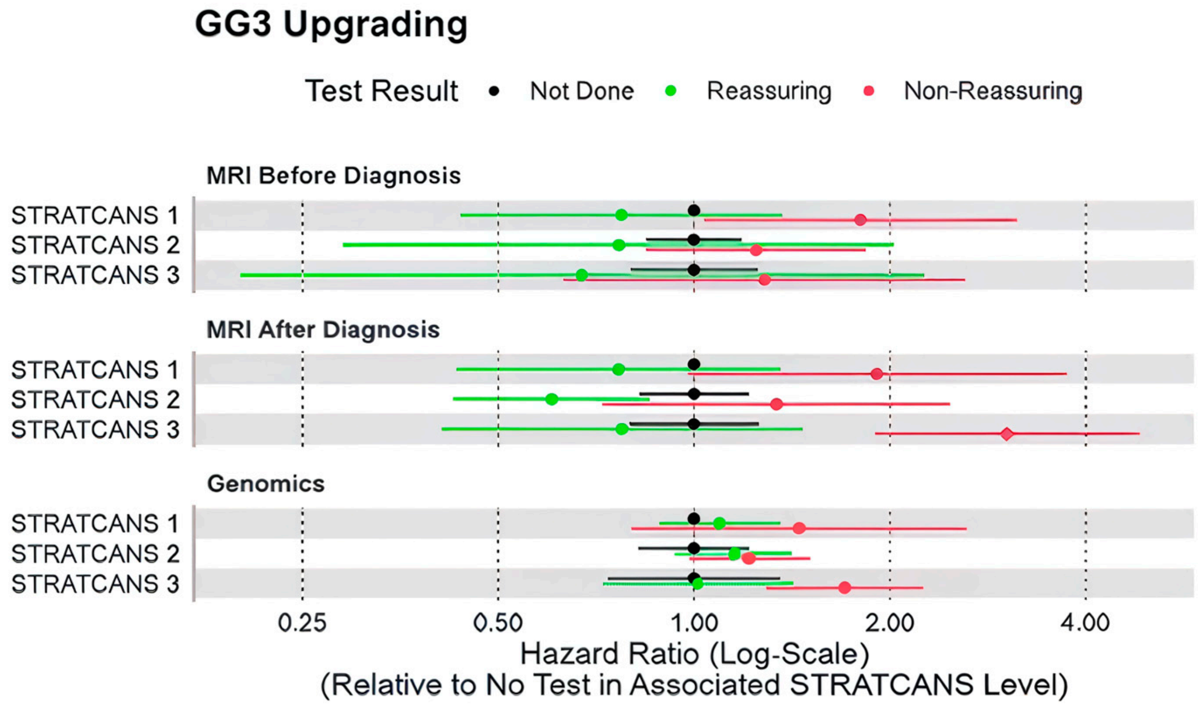


(b)

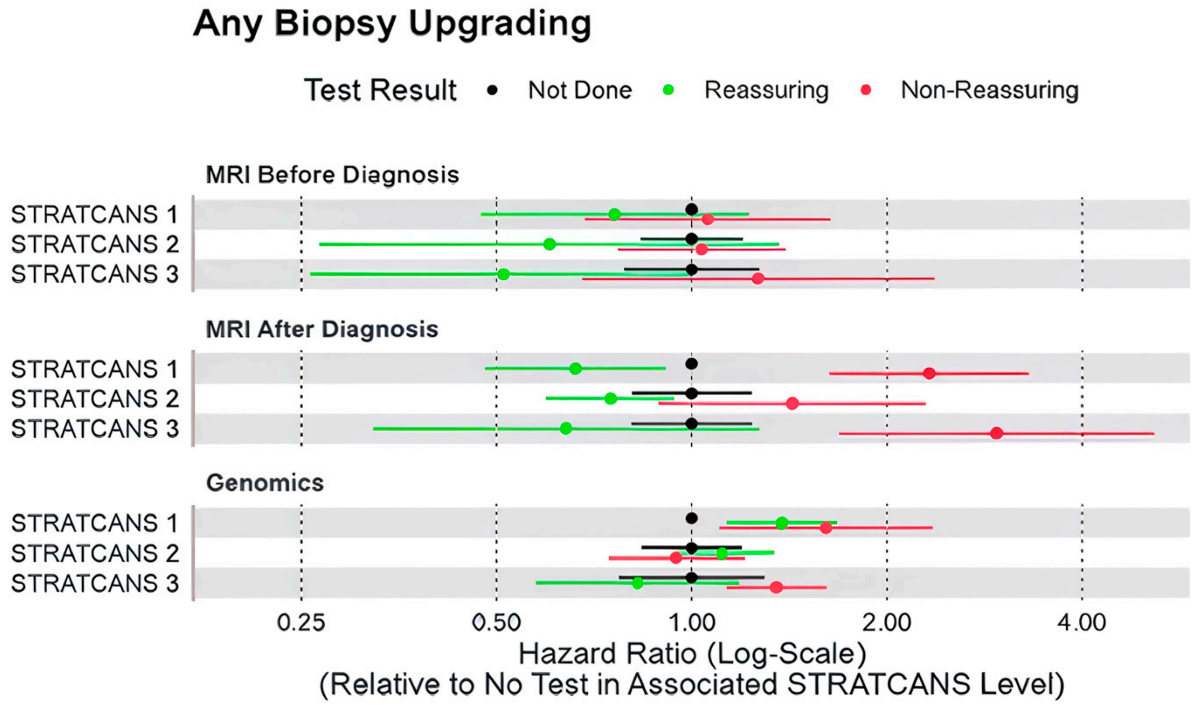

(c)

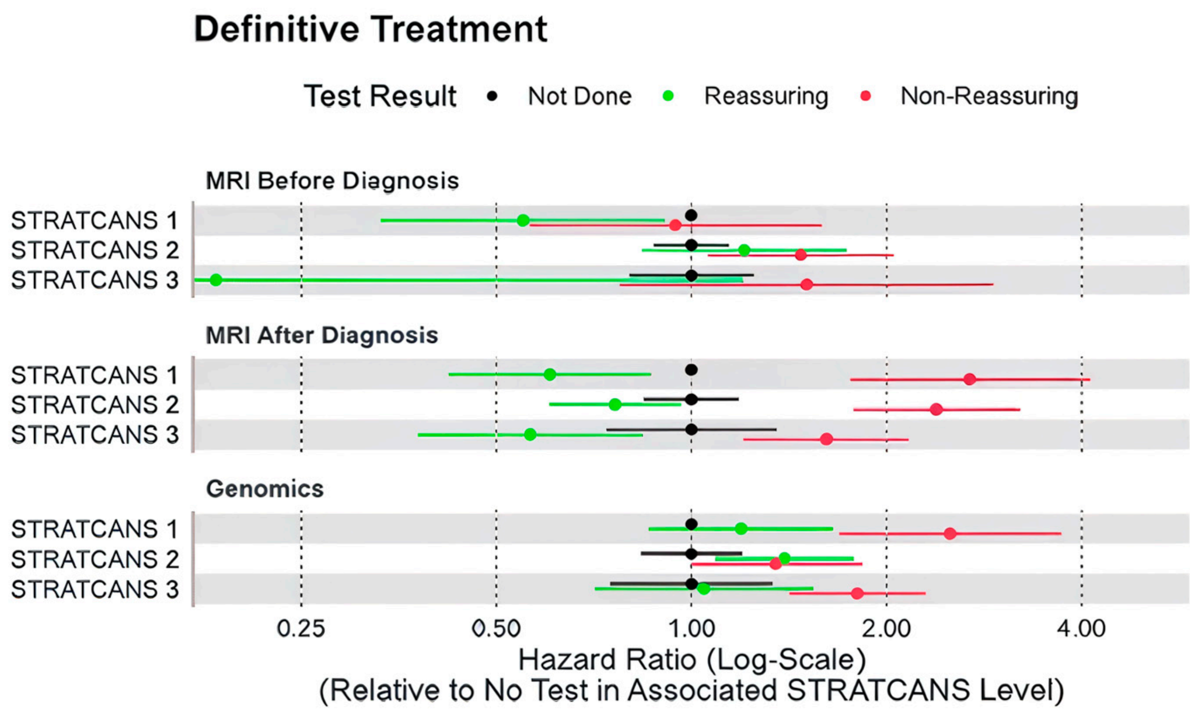


## References


1. Sjoberg DD, Baillie M, Fruechtenicht C, Haesendonckx, S., Treis, T. ggsurvfit: Flexible Time-to-Event s. **2024**. https://cran.r-project.org/web/packages/ggsurvfit/index.html (accessed on 5 September 2025).
2. Therneau TM. coxme: Mixed Effects Cox Models. **2024**. https://cran.r-project.org/web/packages/coxme/index.html (accessed on 5 September 2025).
